# Risk and protective factors of neurocognitive disorders in older adults in Central and Eastern Europe: A systematic review of population-based studies

**DOI:** 10.1371/journal.pone.0260549

**Published:** 2021-11-30

**Authors:** Katrin Wolfova, Matej Kucera, Pavla Cermakova

**Affiliations:** 1 Department of Psychiatry and Medical Psychology, Third Faculty of Medicine, Charles University, Prague, Czech Republic; 2 National Institute of Mental Health, Klecany, Czech Republic; 3 Department of Epidemiology, Second Faculty of Medicine, Charles University, Prague, Czech Republic; Cardiff University, UNITED KINGDOM

## Abstract

**Background:**

A wide range of potentially modifiable risk factors, indicating that the onset of neurocognitive disorders can be delayed or prevented, have been identified. The region of Central and Eastern Europe has cultural, political and economic specifics that may influence the occurrence of risk factors and their link to the cognitive health of the population.

**Objective:**

We aimed to systematically review population-based studies from Central and Eastern Europe to gather evidence on risk and protective factors for neurocognitive disorders.

**Methods:**

We searched the electronic databases PubMed, Cochrane Database of Systematic Reviews, PsycINFO, Web of Science, and Embase. The search was performed on 26^th^ of February 2020 and repeated at the end of the review process on 20^th^ May 2021.

**Results:**

We included 25 papers in a narrative synthesis of the evidence describing cardiovascular risk factors (n = 7), social factors (n = 5), oxidative stress (n = 2), vitamins (n = 2), genetic factors (n = 2) and other areas (n = 7). We found that there was a good body of evidence on the association between neurocognitive disorders and the history of cardiovascular disease while there were gaps in research of genetic and social risk factors.

**Conclusion:**

We conclude that the epidemiological evidence from this region is insufficient and population-based prospectively followed cohorts should be established to allow the development of preventive strategies at national levels.

## Introduction

The demographic shifts and associated needs of the ageing populations bring significant challenges to society and health care systems. Neurocognitive disorders, which encompass a wide range of conditions, including mild cognitive impairment and several types of dementia, carry perhaps the greatest social and healthcare costs of the demographically ageing populations. Decline in cognitive functions, which refer to the ability to remember, reason, learn, and think clearly, is the main driver of the costs associated with impairment in independent living [1, 2], as well as a risk factor for mortality, disability, and poor quality of life [3–6]. Currently, there is no cure for neurocognitive disorders, therefore, the importance of their prevention is paramount [1, 7]. The World Health Organization (WHO) claims dementia a public health priority and encourages individual countries to formulate detailed policies addressing needs of this population and endorsing prevention [1, 8].

Age is the main risk factor for cognitive dysfunction but not all older adults experience neurocognitive disorders. Common cardiovascular risk factors, including hypertension, diabetes mellitus, physical inactivity, smoking, hyperlipidemia and obesity, are strongly related to the risk of neurocognitive disorders [9–11]. A wide range of psychosocial characteristics was also proposed as putative risk factors, including social isolation, low work complexity and control, high workload, psychological stress, anxiety or depression [12]. The risk of neurocognitive disorders is also shaped by social structural factors: high socioeconomic position, reflected in markers such as education, occupation and material circumstances, is associated with lower risk of developing dementia in a graded manner [13]. The existence of potentially modifiable risk factors indicates that the onset of neurocognitive disorders can be delayed and/or prevented. Projections based on US data show that a delay of dementia onset by 1 year will reduce the population aged 70+ with dementia in 2030 from 5.8 to 4.7 million, and in 2050 from 9.1 to 7.8 million [14].

Population-based studies conducted during the past decade suggest declining dementia incidence [15, 16]. No single protective or risk factor can explain these trends [15, 16], but in particular, increase in years of education, improved control of cardiovascular risk factors and decreasing occurrence of cardiovascular diseases likely contributed to these positive changes. This indirectly adds to the evidence that prevention of neurocognitive disorders is possible. In 2019, WHO published guidelines for the “Risk reduction of cognitive decline and dementia” and encouraged countries to adapt them to their local contexts before implementing strategies to reduce the risk factors for neurocognitive disorders. However, much of the epidemiological evidence about risk factors for neurocognitive disorders as well as trends in their occurrence come from countries situated in the US and Western Europe, while evidence regarding neurocognitive disorders from non-western settings is rare [17].

Even in Europe, there is a major gap in knowledge; it was noted that data on trends in cognitive health from Central and Eastern Europe (CEE) are largely lacking [15, 16]. The region of CEE has a culturally, politically and economically specific context that may influence cognitive health of the population. While cardiovascular morbidity and mortality have been declining since the 1970s in Western Europe, the rates have been increasing in CEE during the second half of the 20^th^ century [18, 19], possibly as a consequence of socioeconomic disruptions caused by the communist regime [18–22]. After the fall of the Berlin wall, many CEE countries underwent a social and economic transition into westernized societies. Recent studies suggest improvements in the control of cardiovascular risk factors and declining mortality from cardiovascular diseases in some CEE countries [23–25]. The improvement in health following the fall of communism led to an increase in life expectancy and as a result, CEE countries face challenges of increasing prevalence of age-related neurocognitive disorders.

Despite specific needs of older adults associated with their declining cognitive functions, CEE countries have been underrepresented in research on cognitive disorders [26, 27], possibly due to limited research infrastructure and reduced research capacities. Furthermore, there is great heterogeneity between individual countries with regard to their health as well as the amount and quality of evidence. A lack of local epidemiological evidence is an important obstacle in creating public policy plans for the prevention of neurocognitive disorders. Due to different risk profiles of the population, it cannot be assumed that interventions that prove effective and feasible in western settings could help citizens in CEE. The aim of our study is to evaluate the extent of evidence about this topic in CEE by conducting a systematic review on risk and protective factors for neurocognitive disorders in older adults in CEE based on evidence from population-based studies.

## Methods

The study protocol was registered in PROSPERO Prospective Register of Systematic Reviews (CRD42020166886).

### Search strategy

We searched the following electronic databases: PubMed, Cochrane Database of Systematic Reviews, PsycINFO (Ovid), Web of Science, and Embase (Ovid). We used a key term composed of 4 components: diagnosis (MeSH terms: neurocognitive disorders OR Keywords: dementia OR Alzheimer disease OR vascular dementia OR cognitive dysfunction OR cognitive impairment OR mild cognitive impairment OR cognitive decline OR cogn*) AND design (MeSH term: observational study OR meta-analysis OR systematic review OR Keywords: population-based study OR population study OR community-based study OR epidemiology OR epidemiological study) AND risk and protective factors (MeSH terms: risk factors OR protective factors OR Keywords: cardiovascular OR heart failure OR hypertension OR high blood pressure OR hyperlipidaemia OR hypercholesterolemia OR high cholesterol level OR smoking OR obesity OR high BMI OR lack of exercise OR physical inactivity OR diabetes OR hyperglycaemia OR high blood sugar OR atherosclerosis OR apolipoprotein E4 OR genetic OR social risk factors OR socioeconomic risk factors OR education OR socioeconomic position OR socioeconomic status OR occupation OR job OR work OR marital status OR number of children OR depress* OR head injury OR craniocerebral trauma) AND countries (Keywords: Albania OR Bulgaria OR Croatia OR Czech Republic OR Czechia OR Hungary OR Poland OR Romania OR Slovak Republic OR Slovakia OR Slovenia OR Estonia OR Latvia OR Lithuania). The search was performed on 26^th^ of February 2020. We didn’t impose any restrictions in terms of publication date or language. At the end of the review process we repeated the search covering the period from 26^th^ Feb 2020 to 20^th^ May 2021 to include any newly published studies. We identified 2 additional relevant articles during the second search. We checked all relevant retrieved systematic reviews to identify potentially missed studies.

### Inclusion and exclusion criteria

We included population-based studies examining risk factors for neurocognitive disorders such as dementia, Alzheimer’s disease, vascular dementia and mild cognitive impairment diagnosed by a healthcare specialist, or studies examining the level of cognitive impairment, level of cognitive functions or rate of cognitive decline. In addition, we included studies examining the prevalence of the above-mentioned disorders as well as dementia mortality rate. Population-based studies were defined as studies with a random sampling method regardless of response rate and loss to follow-up. The study populations were restricted to middle-aged and older adults including but not limited to participants 50+ age at baseline; younger cohorts were also included if they were followed up in time and they reached the age of at least 50 years during the time of the follow-up. Only studies conducted in countries of CEE defined by OECD were included, i.e. Albania, Bulgaria, Croatia, the Czech Republic, Hungary, Poland, Romania, the Slovak Republic, Slovenia, Estonia, Latvia and Lithuania [28]. Studies with participants from CEE that were part of a multinational consortium were also included. We excluded studies focused on conditions such as Parkinson’s disease without information on cognitive functioning, Huntington’s disease, multiple sclerosis and early-onset dementia. Next, we excluded studies with outcomes defined as biomarkers or morphologic correlates. Conference abstracts were not included.

### Procedures

All references were imported into EndNote for de-duplication and then into Rayyan for review [29]. A pilot set of 100 abstracts was independently reviewed by 2 reviewers (KW, MK) according to the listed inclusion and exclusion criteria. Reviewers involved in the pilot stage met to assess and resolve any disagreements on classification and refined and standardized the inclusion and exclusion criteria. The registered protocol was updated. The refined inclusion and exclusion criteria were used to evaluate the remaining abstracts for the first round of review. In the second round of the review, the full texts were retrieved. An assessment of the risk of bias was conducted. Data from all included studies were extracted into a purpose-built database. Disagreements were resolved by discussion with a third review author (PC).

### Assessing the risk of bias

The risk of bias was assessed using the Joanna Briggs Institute Critical Appraisal Checklist for Studies Reporting Prevalence Data [30]. Each domain was reviewed independently by two authors (KW, MK) as recommended by the PRISMA guidance [31]. The checklist consists of 9 questions with four possible answers: yes, no, unclear, and not applicable. The risk of bias was considered “high” when up to 49% of the answers corresponded to "yes", “moderate” when 50% to 69% of the answers corresponded to "yes", and “low” when more than 70% of the answers corresponded to "yes". The review of ratings and final decision over bias classification was made by the third author (PC).

## Results

### Study selection

**Fig 1:**
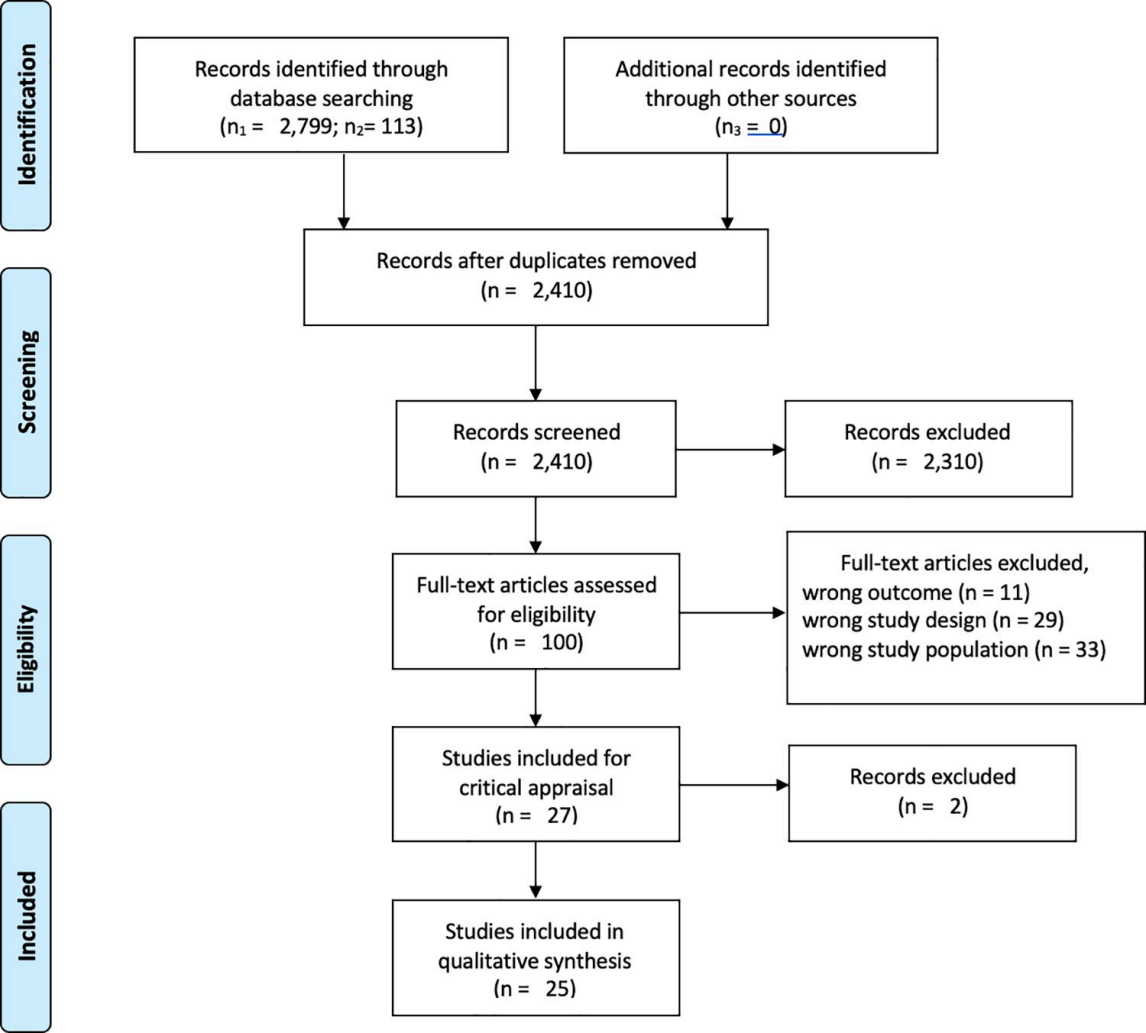
PRISMA flow-chart.

A total of 2,799 articles were retrieved from the initial database search, of which 2,299 articles remained after duplicates were removed. Next, article titles and abstracts were screened for eligibility leaving a total of 100 articles. In the final step, full text articles were screened, which resulted in 26 studies left in the sample. The second round of database search resulted in the retrieval of 113 articles, with 111 remaining after removing duplicates, and 2 were identified as relevant. No additional articles were identified in literature during the review process. This resulted in inclusion of 28 studies meeting the eligibility criteria. Finally, 2 studies met criteria for high risk of bias and are not included in the review. Their characteristics are in S1 Table. The PRISMA flow-chart presenting the selection process is included in Fig 1.

### Characteristics of the studies

The characteristics of 25 included studies with low risk of bias are presented in Table 1. A total of 20 studies were cross-sectional, 5 were prospective. Sample sizes in included studies ranged from 270 to 25,127. In prospective studies, the follow-up period ranged from 3.7 to 5.5 years. A total of 16 studies were conducted only on a sample from one CEE country (10 in Poland, 3 in the Czech Republic, 1 in Croatia, 1 in Lithuania, 1 in Bulgaria). In the rest of the studies (n = 9), a country from CEE region was part of a multinational sample. Specifically, those analyses were derived from datasets based on data from the Survey of Health, Ageing, and Retirement in Europe (SHARE), Health, Alcohol and Psychosocial Factors in Eastern Europe (HAPIEE) and European Male Ageing Study (EMAS). We have not identified any study that would include participants from Albania, Romania, the Slovak Republic and Latvia and that would meet our inclusion criteria.

**Table 1:** Characteristics of selected studies.

Author (year)	CEE region represented by	Project	Type of study	Follow-up (if applicable)	Sample size	Response rate	Male	Age (years)	Dementia / cognition assessment
Cardiovascular risk factors									
Pajak A. et al. (1998)	Poland	CASCADE Krakow	cross-sectional	-	882	94%	42%	65–78	MMSE
Tamosiunas A. et al. (2012)	Lithuania	HAPIEE	cross-sectional	-	6,904	64.8%	45.3%	Men: lowered CF 61.0 ± 0.3, normal CF: 60.4 ± 0.15	immediate verbal recall, delayed verbal recall, verbal fluency, cognitive speed and concentration (letter cancellation), numerical ability
								Women: lowered CF 60.4 ± 0.35, normal CF 60.3 ± 0.13	
Horvat et al. (2015)	Czech Republic, Poland	HAPIEE	prospective	baseline: 2002–2005	14,575	59% at baseline;	45.3%	47–78	immediate verbal recall, delayed verbal recall, verbal fluency, cognitive speed and concentration (letter cancellation)
				Follow-up: 2006–2008		63% at follow-up			
Kostka T. (2008)	Poland	-	cross-sectional	-	270	39.6%	58.8%	Men: 69.8 ± 4.0	MMSE
								Women: 70.0 ± 4.1	
Puzianowska-Kuznicka M. et al. (2019)	Poland	PolSenior Study	cross-sectional	-	4,944	95.7%	51.7%	-	MMSE
Turnoy et al. (2010)	Poland, Hungary, Estonia	EMAS	cross-sectional	-	3,152	41%	100%	Absent metabolic syndrome: 59.3 (11.2)	Rey–Osterrieth Complex Figure test, the Camden Topographical Recognition Memory test and the Digit Symbol Substitution Test
								Present metabolic syndrome: 61.0 (10.4)	
Szcześniak D. et al (2021)	Poland	PURE-MIND	cross-sectional	-	547	64%	35.6%	Mean 56.2 ± 6.5	The Montreal Cognitive Assessment (MoCA): score below 26 points was defined as mild cognitive impairment; Digit Symbol Substitution Test; Trail Making Test
Social factors									
Cermakova et al. (2018)	CEE (countries not specified)	SHARE	prospective	Average follow up 4.8 years with an SD of 3.1 years	CEE: 5,039	Ranged from 54% to 62% at baseline;	46%	median at baseline: 71	verbal learning and delayed recall (gained from an adapted 10-word delay recall test) and verbal fluency (from an animal word fluency test)
					Whole sample: 20,244	73% retention rate during follow-up			
Horvat et al. (2014)	Poland, Czech Republic, Lithuania	HAPIEE	cross-sectional	-	25,127	59%	45%	Mean 60	Word recall task, verbal fluency, letter cancellation
Bjelajac A. K. et al. (2019)	Croatia	SHARE	cross-sectional	-	650	42%	50.9%	Men: median 57 (IQR 55–60)	Numeracy (calculating with percentages, simple subtraction), verbal fluency, and verbal recall (immediate and delayed)
								Women: median 55 (IQR 53–58)	
Stepankova Georgi H. et al. (2019)	Czech Republic	NANOK	cross-sectional	-	324	-	Prague: 46%	Mean 68.06 ± 3.08	MMSE, Story, Trail Making Test, Digit Symbol Substitution Test, Prague Stroop Test: dots (PST-D), neutral words (PST-W), colors (PST-C), Category verbal fluency test–animals, Rey auditory-verbal learning test–trial 1, Simple Reaction Time task, Go/No-Go task
							Towns: 49%		
							Villages: 50%		
Tosheva, E.	Bulgaria	SHARE	Cross-sectional	-	2001	-	-	Persons aged 55 and more	immediate word recall, delayed recall
Vitamins									
Horvat P. et al. (2016)	Poland, Lithuania, Czech Republic	HAPIEE	prospective	Average follow up 3.8 years with an SD of 0.4 years (range 1.8–5.5)	Cross-sectional: 4,166Prospective: 2,739	-	66%	Cross-sectional: mean 64.5Prospective: mean 64.7 at baseline and 68.4 at re-examination	immediate word recall, delayed recall, verbal fluency, timed letter search
Lee D.M. et al. (2009)	Poland	EMAS	cross-sectional	-	3,133	-	100%	Mean 59.9 ± 11.0	Rey–Osterrieth Complex Figure (ROCF) test, Camden Topographical Recognition Memory (CTRM), Digit-Symbol Substitution Test (DSST)
	Hungary								
	Estonia								
Oxidative stress									
Horvat P. et al. (2016)	Poland, Czech Republic, Lithuania	HAPIEE	prospective	Average follow up 3.7 ± 0.4 years	cross-sectional: 4,304	61% in Poland, 65% in Lithuania, and 55% in the Czech Republic	65.9–67.7%	mean 63.9 at baseline	immediate word recall, delayed recall, verbal fluency, letter cancellation test
					prospective: 2,882				
Bednarska-Makaruk M. et al. (2015)	Poland	PolSenior Study	cross-sectional	-	3,154	42%	49.8%	mean 76.3	MMSE
Genetic factors									
Golanska E. et al. (2013)	Poland	-	cross-sectional	-	centenarians: 150,	centenarians: 13.3%	93%	centenarians: 101.1 ± 1.1	diagnosis of dementia by DSM IV, NINCDS/ADR DA ICD, the clinical dementia rating (CDR) score of 1 or above; or the MMSE score
					controls: 165	controls: 44.8%		controls: 27.8 ± 9.1	
Golanska E. et al (2013)	Poland	-	cross-sectional	-	centenarians: 150,	centenarians: 13.3%	93%	centenarians: 101.1 ± 1.1	diagnosis of dementia by DSM IV, NINCDS/ADR DA ICD, the clinical dementia rating (CDR) score of 1 or above; or the MMSE score
					controls: 165	controls: 44.8%		controls: 27.8 ± 9.1	
Other									
Bdzan L. et al. (2007)	Poland	-	cross-sectional	-	Phase I: 1,000	-	37%	men: mean 69.49	MMSE
									ICD-10 diagnostic criteria for Alzheimer’s disease and vascular dementia
					Phase II: 76			women: mean 72.20	
Klich-Raczka A. et al. (2014)	Poland	PolSenior Study	cross-sectional	-	5,219	42%	65+: 52.1%	men: mean 79.0 ± 8.4	MMSE;
							55–59: 46.1%	women: mean 78.5 ± 8.7	score below 24 points classified as impaired cognition
Pac A. et al (2019)	Poland	PolSenior Study	cross-sectional	-	4,653	42%	51.8%	Mean 79.4 ± 8.72	MMSE; results of 24 points or higher indicate no cognitive impairment
Formanek et al. (2019)	CEE (Czech Republic, Poland, Slovenia, Estonia)	SHARE	prospective	Average follow up 4.8 years with an SD of 3.1 years	CEE: 5,178	Ranged from 54% to 62% at baseline;	46%	median at baseline 71 (IQR 8)	verbal learning and delayed recall (gained from an adapted 10-word delay recall test) and verbal fluency (from an animal word fluency test)
					Whole sample: 22,181	73% retention rate during follow-up			
Bobak M. et al. (2009)	Czech Republic	HAPIEE	cross-sectional	-	Czech Republic: 3,626	Czech Republic: 55%	Czech Republic: 45.1%	-	immediate word recall, verbal fluency, timed letter search
					Russia: 3,874	Russia: 61%	Russia: 47.4%		
Seblova D. et. al (2019)	Czech Republic	SHARE	Repeated cross-sectional	-	Cohort 1 (wave 2): 1,071	Cohort 1: 60%	Cohort 1: 40%	Cohort 1: median 73 (10)	verbal fluency, immediate recall, delayed recall, and temporal orientation
					Cohort 2 (wave 6): 2,980	Cohort 2: 89% (retention rate)	Cohort 2: 43%	Cohort 2: median 72 (10)	
Lee D. M. et al. (2009)	Poland, Hungary, Estonia	EMAS	cross-sectional	-	3,265	Ranged from 24% in Szeged to 62% in Florence	100%	Mean 59.9 ± 10.9	Rey–Osterrieth Complex Figure, Camden Topographical Recognition Memory, Digit-Symbol Substitution

*Note*. CASCADE—Cardiovascular Determinants of Dementia, EMAS–European Male Ageing Study, HAPIEE–Health, Alcohol and Psychosocial factors In Eastern Europe, NANOK study–National Normative Study of Cognitive Determinants of Healthy Ageing, SHARE–Survey of Health, Ageing and Retirement in Europe.

Cardiovascular risk factors were examined in 7 studies focused on the history of cardiovascular diseases as well as factors that are known to increase cardiovascular risk such as obesity, metabolic syndrome, hyperglycaemia, hypertension, high plasma lipid levels, smoking or alcohol consumption. A total of 4 studies focused attention on the effect of risk factors occurring in childhood such as low socioeconomic status and education. There was 1 study with a focus on social engagement. The effect of oxidative stress on cognitive functions was addressed in 2 studies. There were 2 studies investigating the relationship between levels of vitamins and cognitive performance in later life. The effect of genetic factors on cognition was assessed in 2 studies. In addition, there were 7 studies focused solely on prevalence data or between-countries comparisons.

A common cognitive outcome was Mini Mental State Examination (MMSE) score (used in 6 studies), where cognitive impairment was additionally defined as MMSE lower than 24 points in 2 studies. The diagnosis of dementia by a physician appeared in 1 study. In 3 studies, cognitive performance was measured using Rey–Osterrieth Complex Figure test, the Camden Topographical Recognition Memory test and the Digit Symbol Substitution Test. The majority of the remaining studies utilized composite scores of multiple cognitive tests on immediate and delayed verbal recall, verbal fluency, Montreal Cognitive Assessment (MoCA), cognitive speed, concentration, numerical ability, temporal orientation, visual search, processing speed and visuo-graphomotor control.

### Cardiovascular risk factors

Two studies based on data from HAPIEE cohort focused on cardiovascular risk factors related to cognition. In the first one, which included a sample only from Lithuania, significantly higher odds of lowered cognitive functions were estimated in men and women with a history of previous stroke and men with ischemic heart disease [32]. Lowered cognitive functions were more frequent in men than women. In the second one, Horvat et al. found substantial sex differences in the relationship between alcohol consumption and cognition in a sample of participants from the Czech Republic, Poland and Russia [33]. After adjusting for socioeconomic and lifestyle confounders, they did not find any association between higher average alcohol consumption or higher drinking frequency and cognitive functions in men cross-sectionally or prospectively. Those who identified themselves as non-drinkers had lower cognitive scores and those who stopped drinking during follow-up period had worse cognitive functions than stable drinkers.

In a study from Poland based on data from the Cardiovascular Determinants of Dementia (CASCADE) project, the authors found that history of previous myocardial infarction was not associated with cognitive impairment [34]. History of previous myocardial infarction as well as presence of severe cognitive impairment (Mini-Mental State Exam (MMSE) < or = 21 points) was found in 15% of participants. Another study from Poland based on a sample of 270 community-dwelling individuals focused on correlates of plasma fibrinogen levels in older adults [35]. No association was revealed in the relationships between plasma fibrinogen levels and the cognition measured by MMSE in the whole study sample, nor in women and men separately. Finally, Szczesniak et. al conducted an analysis based on PURE-MIND data and found a positive association of diabetes, hypertension and obesity with cognitive performance measured by MoCA, Digit Symbol Substitution Test (DSST) and Trail Making Test (TMT) [36]. Coronary heart disease and smoking was associated with lower scores in the DSST task but not with cognitive performance measured by MoCA and TMT. Abstinence from alcohol was associated with lower scores in MoCA. They did not find any association between hyperlipidaemia and cognitive performance. Cerebral small vessel disease, hypertension, depressive symptoms, overweight and obesity increased the probability of mild cognitive impairment (MCI). On the other hand, participants who reported alcohol consumption and those who never smoked had lower likelihood of MCI, and no association was found with hyperlipidaemia, diabetes and coronary heart disease.

An analysis of data from PolSenior Study revealed a seemingly counter-intuitive relationship between body measurements and cognitive functions in later life [37]. Better cognitive performance was associated with higher median waist circumference and arm circumference both in women and men while higher median body mass index (BMI) was associated with better cognition only in women. After adjustment for age and smoking, all three measurements were associated with better scores in cognition in women and BMI and arm circumference were significant predictors in men.

Another cross-sectional study investigated an association of cognition with metabolic syndrome and with glycaemia only in men from Poland, Hungary and Estonia together with other nations (Italy, Belgium, Sweden, UK, Spain) [38]. When adjusting for age, they found an association between metabolic syndrome and some measures of cognitive functions. The associations lost statistical significance after adjustment for lifestyle and health-related characteristics. However, in additional analysis of individual components of metabolic syndrome, an association between higher glucose levels and lowered cognitive functions was observed.

### Social factors

CEE region was part of an analysis that used data from the multinational SHARE project [26]. In the whole sample, even after adjustment for a broad range of health-related and sociodemographic parameters, poor childhood socioeconomic position was associated with lower cognitive scores at baseline. There was no association between childhood socioeconomic hardships and the rate of cognitive decline during follow-up. Another analysis of individuals from the HAPIEE cohort showed a significant association of socioeconomic position with mid-life and later-life cognition in the whole sample [39]. Using structural equation modelling, the authors found that the association between higher incomes and education was weak and the indirect effect of education on cognition was small. However, education showed the strongest direct path of all the life-course measures related to socioeconomic status.

Klich-Rączka et al. found a decreasing gradient of cognitive impairment with increasing length of education in Poland, ranging from 48.4% among participants with one to six years at school to 3.3% among those with the highest education in unadjusted models and from 36.7% in self-taught participants through 18.7% of those with one to six years of education to 14.5% in the most educated [40]. Results from another study from Poland showed an association of higher secondary education level with higher cognitive functioning (MoCA, DSST, TMT) but no significant difference between people with education at primary level and vocational level was identified [36]. An analysis of a Croatian population derived from the SHARE study focused on a predictive value of employment status and other sociodemographic characteristics, especially in the context of residency area [41]. Regardless of other factors, living in an urban area was associated with better verbal fluency. Urban women scored lower in both numerical tasks than urban men, while in rural subsample women performed worse only in subtraction. Additionally, living with a partner had a protective effect on cognition, while unemployment predicted the opposite. Higher education was identified as a protective factor for cognitive functions, regardless of gender and type of residence.

A study from the Czech Republic suggested that living in a big city is associated with higher cognitive performance only in some domains [42]. They found better memory, attention and verbal control performance in the citizens of Prague in comparison to other areas (towns, villages). On the other hand, they did not observe any association between the type of settlement and visual search, processing speed and visuo-graphomotor control.

Finally, one study from Bulgaria reported statistically significant relationship between participation in social activities and cognitive performance measured by immediate recall in older adults [43].

### Oxidative stress

One study explored the link between the presence of blood-based oxidative stress markers and domain-specific cognitive performance in CEE countries [44]. From the measured values of oxidative stress markers, a statistically significant inverse association of derivatives of reactive oxygen metabolites with global cognition and verbal fluency was found in cross-sectional as well as prospective analysis. Total thiol levels were inconsistently associated with memory, as cross-sectional results contradicted prospective associations. No association with cognitive functions has been found for biological antioxidant potential.

Another study suggested an important role of oxidative stress, as oxidized LDL-paraoxonase 1 (PON1) activity was decreased in person with impaired cognition, both in males and females, in a large group of older adults in Poland [45]. On the other hand, no relationship was found in case of the level of anti-oxidized LDL antibodies. The role of PON could be explained by concurrently higher measurements of inflammation indicators.

### Vitamins

A study based on sample from HAPIEE cohort provided inconsistent evidence on the connection between serum folate and vitamin B-12 concentrations and cognitive performance [46]. There were positive associations between serum folate and B12 concentration and some cognitive functions. However, a prospective analysis showed steeper decline in immediate recall associated with lower folate concentrations, while better verbal fluency was associated with both higher serum folate and B-12. Another study assessed the relation between 25-hydroxyvitamin D levels and cognitive performance in men from EMAS [47]. Adjusting for age, higher levels of vitamin D were associated with better scores in cognitive tests. After adjustment for confounders, the association to the majority of cognitive tests lost statistical significance.

### Genetic factors

Two studies conducted in Poland explored the relationship between genetic risk factors with cognitive outcomes. In the first one, the authors investigated the polymorphism of the APBB2 gene, of which the over-expression promotes the form of β-amyloid, a major component of senile plaques, and thus could play a significant risk factor in the development of cognitive decline at a later age [48]. The authors identified an increased frequency of the APBB2 rs13133980G allele in individuals with low cognitive performance. In the second study, they addressed the prion protein M129V polymorphism, which may affect the level of neurodegeneration at a later age [49]. No statistically significant evidence was found that genotypic frequencies differed for the two groups stratified by cognition.

### Other

Results of a Polish study focused on a rural population indicated that the prevalence of dementia is around 6.7% among people 60 years old and older, with worse outcomes for women (3.0% men, 8.8% women) [50]. Prevalence of Alzheimer’s disease was estimated as 1.1% for men and 4.0% for women, vascular dementia 1.9% and 3.5%, respectively. Another study from Poland estimated higher frequencies of suspected dementia based on MMSE score lower than 24 points, again with slightly more prevalent cognitive impairment among women (21%) than among men (19.4%) [40]. Based on a dataset derived from the PolSenior Study, cognitive impairment was estimated to be present in 21.5% of Polish older citizens (men: 20,1%, women: 22,4%) [51]. This study was primarily focused on the concept of healthy ageing based on three operative definitions and no other estimates related to cognition were calculated.

Analysis of data from the Czech and Russian sample derived from the HAPIEE study revealed differences between the two nations in the association of the year of birth with cognitive functioning [52]. Even after adjusting for a wide range of biological and socioeconomic covariates, there was steeper slope of association between the birth year and lower cognitive performance in Russian men and women than in the Czech men and women.

Another study assessed differences in cognitive functions in later life among European regions [27]. At baseline, participants from CEE performed better in cognitive tests than their counterparts from Mediterranean countries but worse than participants from Scandinavia and Western Europe. Together with Mediterranean countries, CEE region was associated with lower overall cognition compared to Western Europe, while higher levels of cognition were associated with living in Scandinavia. On the other hand, CEE region as well as Western Europe and Mediterranean countries had lower rates of annual decline in overall cognition and in separate tests than Scandinavia.

One study based on data from the EMAS assessed the between-centre and within-centre variance of cognitive performance in several cognitive tests [53]. They found a between-centre variance in crude models for all the three tests. Age, full-time education, present depressive symptoms, comorbidities, smoking and alcohol consumption explained between 10 and 13% of the variation in cognitive measures. The same covariates accounted for between 17 and 36% of the within-centre variation in cognitive scores. Participants from CEE scored lower in cognitive tests than participants from Western Europe and Scandinavia, but higher than their counterparts from Southern Europe.

Finally, one study suggested that the age-specific prevalence of cognitive impairment in the Czech Republic decreased over a period of 9 years [54]. The authors compared age-specific prevalence in two cohorts from the SHARE project (wave 2: 2006/2007 vs. wave 6: 2015) using four different definitions to indicate cognitive impairment. The cognitive impairment in cohort 2 was 1.2 to 2.3 times lower than in cohort 1, based on different definitions. The results from multivariate decomposition suggested that the prevalence differences could be attributed to differences in cohort characteristics such as physical inactivity, management of high blood cholesterol, decreased occurrence of stroke and increased length of education.

## Discussion

Contributions of non-modifiable risk factors such as genetics to dementia are substantial, however, an evidence synthesis report of the Lancet commission from 2020 states there are 12 potentially-modifiable risk factors occurring during childhood (less education), midlife (hypertension, obesity, hearing loss, traumatic brain injury, and alcohol misuse) and later life (smoking, depression, physical inactivity, low social contact, diabetes and air pollution) [7]. Nevertheless, almost all the evidence is from high-income Western countries and the risk in CEE might significantly differ, as there are great differences in socioeconomic development and a large gap in cardiovascular health between this region and Western countries. Some CEE countries have already adopted national dementia plans, but the majority have yet to do so [55]. To ensure actions taken at the national level are effective, planned interventions should be modified to fit cultural and environmental specifics. Our aim was to gather evidence on risk and protective factors for neurocognitive disorders coming from CEE.

We identified only a small number of studies in CEE that implemented random sampling methods and only few of them were prospective. Even in those studies, the response rate was often low and the attrition high. This limits the generalizability of their findings and potentially underestimates the associations of risk factors to cognitive functioning. In addition, due to the short follow-up period in prospective studies, relying largely on samples from older adults, selective survival bias precludes from drawing causality. The majority of studies conducted in CEE suggested similar risk factors for neurocognitive disorders that were found in western setting. There was a body of evidence on the association between neurocognitive disorders and history of cardiovascular disease, even though one of the smaller studies from Poland did not find any association between myocardial infarction and cognitive impairment. On the other hand, the role of individual cardiovascular risk factors is less clear. Hypertension and smoking were identified as risk factors in one study and there was a trend for hypercholesterolemia as well. In addition, there were two contra-intuitive studies suggesting that alcohol and higher BMI might have protective effect on later-life cognition.

Studies evaluating cognitive differences between urban and rural populations provided information on slightly better cognitive outcomes for people living in the cities. However, we identified large gaps in research of social risk factors in the region of CEE as the role of low social contact or depression has not been explored. In addition, there were two studies focused on vitamins and two studies examining oxidative stress–factors that might not play as important role, while the contribution of hearing loss, traumatic brain injury, physical inactivity, or air pollution has not been explored in any of the included studies.

There were only two studies from Poland examining genetic risks on a population level, specifically polymorphism of the APBB2 gene and prion protein M129V. The major risk allele for dementia is APOE4 and many studies from Western countries have assessed its impact on its population [56]. Even though genetic factors are non-modifiable, the contribution of APOE genotype to the overall risk should be estimated also in CEE countries as the results from Western world might not apply to other ethnicities.

The research area of risk and protective factors for neurocognitive disorders remains largely unexplored in the region of CEE, with a lack of nationally representative longitudinal studies. The epidemiological evidence is insufficient to guide the content of intervention trials and preventive strategies at the national levels. Future efforts should be focused on the establishment of population-based prospectively followed cohorts, ideally as a part of an international consortium to allow between-nation comparisons.

## Supporting information

S1 Checklist PRISMA 2020 checklist. (DOCX)

S1 Table Studies not included in the review due to high risk of bias. (DOCX)
