# Supplementary material for: Risk and protective factors of neurocognitive disorders in older adults in Central and Eastern Europe: A systematic review of population-based studies
Source: PLoS One. 2021 Nov 30;16(11):e0260549. doi: 10.1371/journal.pone.0260549 (PMC8631612; doi:10.1371/journal.pone.0260549)
Supplement: S1 Table — (DOCX) [file pone.0260549.s002.docx]

Risk and protective factors of neurocognitive disorders in older adults in Central and Eastern Europe: a systematic review of population-based studies

Short title: Cognitive aging in Central and Eastern Europe: a systematic review

Katrin Wolfova^1,2,3^, Matej Kucera^2,3^, Pavla Cermakova^2,3^

*^1^ Department of Psychiatry and Medical Psychology, Third Faculty of Medicine, Charles University, Prague, Czech Republic*

*^2^ National Institute of Mental Health, Klecany, Czech Republic*

*^3^ Department of Epidemiology, Second Faculty of Medicine, Charles University, Prague, Czech Republic*

| **S1 Table. Studies not included in the review due to high risk of bias** | | | | | | | | | |
| --- | --- | --- | --- | --- | --- | --- | --- | --- | --- |
| Article | Author  (year) | CEE region represented by | Type of study | Follow-up (if applicable) | Sample size | Response rate | Male | Age  (years) | Dementia / cognition assessment |
| Education level does not affect prevalence of dementia in a Bulgarian population | Dimitrov I. et al.  (2011) | Bulgaria | Cross-sectional | - | 540 | 89.3% | 41.3% | mean 72.95 | MMSE, dementia diagnosed based on Diagnostic and Statistical Manual of Mental Disorders 4th edition (DSM-IV) criteria |
| Dietary patterns associated with Alzheimer's disease: population based study | Gustaw-Rothenberg, K.  (2009) | Poland | Cross-sectional | - | 71 | - | - | - | Dementia diagnosed by the DSM-IV criteria, Alzheimer’s disease was diagnosed in accordance to the National Institute of Neurological and Communicative Disorders and Stroke/Alzheimer’s Disease and Related Disorders Association (NINCDS-ADRDA) criteria. |
